# Integrin-mediated resistance to epidermal growth factor receptor-targeted therapy: an inflammatory situation

**DOI:** 10.1186/s13058-014-0448-0

**Published:** 2014-09-23

**Authors:** Wells S Brown, Michael K Wendt

**Affiliations:** Department of Medicinal Chemistry and Molecular Pharmacology, Purdue University, West Lafayette, IN 47906 USA

## Abstract

Targeting the function of epidermal growth factor receptor (EGFR) has failed as an effective clinical option for breast cancer. Understanding the drivers of inherent resistance has been a challenge. One possible mechanism is the acquisition of stem-like properties through the process of epithelial-mesenchymal transition. A recent study by Seguin and colleagues adds to our understanding of this process by demonstrating a functional role for unligated αvβ3 integrin in mediating a stem-like phenotype and facilitating resistance to EGFR-targeted therapy via enhanced downstream coupling to a KRAS:RalB:NF-κB pathway. Importantly, the identified mechanism may reveal a possible strategy for sensitizing breast cancer cells to EGFR-targeted therapies.

## Background

Expression of epidermal growth factor receptor (EGFR) serves as a robust marker for the basal-like subtype of breast cancer (BC) and a biomarker for decreased survival times of BC patients [1,2]. Moreover, numerous studies have established functional roles for epidermal growth factor/EGFR in facilitating the systemic dissemination of BC cells [3–5]. However, clinical trials targeting EGFR for the treatment of BC have produced disappointing results [6,7]. These contrasting findings suggest that while BC cells utilize EGFR signaling to facilitate certain aspects of tumor progression, they become inherently resistant to inhibition of EGFR function during the later stages of disease progression. Defining the drivers of this type of inherent resistance has been difficult because, as opposed to acquired resistance, these cells are resistant prior to therapeutic intervention.

Integrin receptors are vital conduits that mediate cell-cell and cell-extracellular matrix interactions, as well as initiating intracellular signaling cascades promoting survival and migration. The complexity of signaling events that emanate from these receptors has prevented effective targeting of their function for the treatment of BC. β3 integrin is a well established marker of highly aggressive and metastatic BC [8]. Furthermore, aberrant expression of β3 integrin is strongly related to epithelial-mesenchymal transition [9], a process involved in several aspects of tumor metastasis [10], generation of a stem-like state [11] and resistance to EGFR-targeted therapies [12]. However, the functional role of β3 integrin in mediating a stem-like state and resistance to EGFR-targeted therapies has remained undefined.

## The article

In a recent article published in *Nature Cell Biology*, Seguin and colleagues [13] demonstrate the importance of an αvβ3:KRAS:RalB:NF-κB signaling pathway in driving a stem-like state and resistance to EGFR-targeted therapies. In this work, the authors first demonstrate that patient-derived tumor tissues can be enriched in tumor-initiating potential based on expression of β3 integrin. Correspondingly, depletion of β3 integrin in tumor cell lines decreases stem-like cells as measured by several assays. Importantly, use of the nonadherent tumor sphere assay was the first indication that the role of β3 integrin in modulating tumor initiating capacity was independent of its role in cell adhesion. Next, the authors demonstrate that β3 integrin is necessary and sufficient to impart growth resistance to the EGFR inhibitor erlotinib. Interestingly, expression of β3 integrin had no impact on the response to nucleoside analogues or DNA crosslinking drugs, suggesting this result is restricted to targeted therapies. Moving downstream of β3 integrin the authors expand upon their previous findings [14] and show that the ability of β3 integrin to facilitate drug resistance does not function through the more traditional focal adhesion complex, but instead via a KRAS:RalB:TBK1:NF-κB pathway that is facilitated by galectin-3. Finally, the authors demonstrate that combining erlotinib with the proteasome inhibitor bortezomib can overcome β3 integrin-mediated resistance to EGFR inhibition (EGFRi).

## Viewpoint

With the enhanced specificity and reduced toxicity of targeted molecular therapies comes the drawback of inherent and acquired resistance. As stated above, several lines of evidence point to EGFRi as an effective therapeutic strategy for BC, but these predications have not been borne out in the clinic. Together with previous findings, the current study suggests that targeting of galectin-3, RalB [15] or TBK1 [16] may actually be capable of inducing sensitivity to EGFRi therapies. Of particular importance is the treatment of triple-negative BCs, which currently lack any effective targeted therapeutic option. With regard to the other BC subtypes, whether this β3:NF-κB mechanism of resistance takes place during acquisition of resistance to HER2-targeted or endocrine-targeted therapies also remains to be determined [17]. Similar to EGFRi, vascular endothelial growth factor (VEGF)-targeted therapy has only had limited success in BC treatment. Several clinical trials currently underway are combining the VEGF-targeted agent bevacizumab with bortezomib, but further investigations into the role of β3 integrin:NF-κB in this resistance process are clearly warranted.

Mechanistically, the finding that β3 integrin induces stemness during the unligated state is very intriguing. Indeed, cilengitide is a cyclic peptide antagonist of αv binding that is currently in clinical trials for the treatment of multiple cancers. However, the current study demonstrates that this compound is unable to target the stem-like cells that drive drug resistance, suggesting it may not be an effective therapy for BC. Specificity remains a major drawback to compounds targeting the NF-κB pathway. Despite this, several ongoing clinical trials are exploring the combination of bortezomib with erlotinib and other targeted therapies [18]. Using β3 integrin as a histological marker, these clinical approaches can be evaluated for eradication of the stem-like cell population in the hopes of unlocking the potential of erlotinib as an effective BC therapy.

## Abbreviations

BC: Breast cancer
EGFR: Epidermal growth factor receptor
EGFRi: Epidermal growth factor receptor inhibition
VEGFR: Vascular endothelial growth factor receptor
